# Isolation, Chemical Characterization, and Antimicrobial Activity of Secondary Metabolites from *Pseudocyphellaria faveolata*

**DOI:** 10.3390/molecules30061368

**Published:** 2025-03-18

**Authors:** Cecilia Rubio, Javiera Ramírez, Caroline Weinstein-Oppenheimer, Tania F. Bahamondez-Canas, Natalia Quiñones

**Affiliations:** 1 Herbario de Líquenes, Escuela de Química y Farmacia, Facultad de Farmacia, Universidad de Valparaíso, Valparaíso 2340000, Chile; cecilia.rubio-@uv.cl (C.R.); javiera.rp6@gmail.com (J.R.); 2 Magíster en Gestión Farmacéutica y Farmacia Asistencial, Escuela de Química y Farmacia, Facultad de Farmacia, Universidad de Valparaíso, Valparaíso 2340000, Chile; 3 Laboratorio de Innovación Terapéutica y Diagnóstico Molecular, Escuela de Química y Farmacia, Facultad de Farmacia, Universidad de Valparaíso, Valparaíso 2340000, Chile; caroline.weinstein@uv.cl; 4 Centro de Investigación, Desarrollo e Innovación de Productos Bioactivos (CInBIO), Universidad de Valparaíso, Valparaíso 2340000, Chile; tania.bahamondez@uv.cl; 5 Laboratorio de Farmacotecnia Antimicrobiana (LADEFAM), Escuela de Química y Farmacia, Facultad de Farmacia, Universidad de Valparaíso, Valparaíso 2340000, Chile

**Keywords:** *Psedocyphellaria faveolata*, physciosporin, antimicrobial properties, antibiofilm

## Abstract

Introduction: Antimicrobial resistance is a global threat, highlighting the urgent need for novel antimicrobial agents. Among the mechanisms of resistance, bacteria can release drug-degrading enzymes and express efflux pumps, as well as grow in protected aggregates known as biofilms. *Pseudomonas aeruginosa* and *Staphylococcus aureus* are among the most prevalent biofilm infections in chronic wounds, respiratory and urinary tract infections, and device-associated infections. *Pseudocyphellaria faveolata* (Delise) Malme is a lichen with metabolites with unexplored antimicrobial potential. Aims: To identify and characterize the major metabolites present in *Pseudocyphellaria Faveolata* and to determine their antimicrobial activity against *Staphylococcus aureus* and *Pseudomonas aeruginosa*. Methods: The molecules were purified by column chromatography and characterized by NMR spectroscopy. The antimicrobial activity of the compounds was determined in terms of proliferation, adhesion, and viability against *P. aeruginosa* and *S. aureus* by the broth microdilution method and crystal violet staining. Viability was determined by the resazurin reduction assay on normal human fibroblasts to determine cytotoxicity over human cells. Results: The major metabolites were spectroscopically characterized and identified as physciosporin and methyl virensate. Physciosporin showed antimicrobial activity on *S. aureus,* with a MIC of 32 μg/mL and MBC of 128 μg/mL, and prevented biofilm formation from 16 μg/mL. Methyl virensate also had antimicrobial activity on *S. aureus* (MIC = 64 μg/mL). None of these metabolites significantly affected *P. aeruginosa* proliferation, viability, or adhesion. Cytotoxicity of physciosporin at 16 ug/mL on normal human fibroblasts was below 20%. Conclusions: This is the first report on the study of the antimicrobial activity of these compounds. Physciosporin showed promising activity in preventing the formation of *S. aureus* biofilms, which are responsible for chronic infections. These findings provide a foundation for exploring the antimicrobial potential of other lichenic depsidones.

## 1. Introduction

Infectious diseases have gained worldwide relevance due to the advent of antimicrobial-resistant microorganisms. In 2021, the World Health Organization (WHO) established a global strategy to reduce the spread of antimicrobial-resistant organisms. In 2017 and 2022, the WHO published two priority pathogen lists for research and development, focusing on 12 bacterial and 19 fungal species, respectively, and in 2024, the bacterial list was updated with more species of pathogens because of the rapid spread of resistance [1,2]. Additionally, bacteria can employ various resistance and virulence mechanisms, including forming biofilms. Biofilms are aggregated bacterial communities encased in a self-produced extracellular polymeric substance (EPS), protecting against harmful substances, immune factors, and adverse environmental conditions [3]. These biofilms are significantly more resistant to antibiotics than free-floating (planktonic) bacteria due to several interrelated factors, such as the limited antibiotic penetration across the EPS and the slow-growing or dormant state of biofilm bacteria, making them less susceptible to antibiotics that depend on active bacterial metabolism. Once established, biofilms are extremely hard to eradicate and are responsible for chronic and persistent infections, such as chronic lung, wound, and urinary infections [4].

Therefore, there is an urgent need to develop new drugs and optimize the use of existing antimicrobials, shifting the focus from solely bactericidal effects to targeting virulence factors, such as biofilm formation [5]. Concerning these needs, the antimicrobial properties of natural compounds from different sources, including microorganisms, algae, plants, and animals, have been investigated [6,7,8]. Lichen species have also been investigated with substantial potential, such as *Alectoria sarmentosa*, *Cetraria pinastri*, *Cladonia digitata*, *Lecanora frustulosa*, and *Ramalina farinacea* [8,9,10].

Lichen or lichenized fungi are mutualistic ecosystems formed by green algae or cyanobacteria (photobiont) and members of fungi kingdom (mycobiont) [11]. Lichen symbiosis is one of the most successful in nature in terms of habitats of colonization. Lichens are found worldwide, including the most desolated regions, such as cold and hot deserts, and in zones of high altitude. This colonization capacity shows that lichens have evolved in terms of chemical and morphological strategies to survive in stressful environments [12]. The ecological success of lichens has been associated with the secondary products they synthesize and accumulate in their thalli [13].

Lichen has been traditionally used in folk medicine to treat wounds, skin disorders, respiratory and digestive issues, and obstetric and gynecological concerns [14,15]. Advances in science have allowed us to study the medicinal properties of lichens and determine the compounds responsible for those properties. In fact, more than 1000 lichen metabolites have been identified [16]. These metabolites have shown anti-inflammatory, analgesic, antipyretic, antibacterial, antifungal, antiviral, and antineoplastic properties [17,18,19,20]. Within these properties, the antimicrobial activities shown by its secondary metabolites stand out [21]. The lichen secondary metabolites depsides and depsidones such as fumarprotrocetraric acid [22], salazinic acid [23], and atranorin [24] have demonstrated great antimicrobial potential against a broad group of bacteria. Even though these manifold activities of lichen metabolites have now been recognized, their therapeutic potential has not yet been fully explored and thus remains pharmaceutically unexploited [18].

The lichen genus Pseudocyphellaria is included in the Lobariaceae family, along with *Sticta* and *Lobaria*. It receives its name from the absence of cortex (covering the thallus) and its interruption-generating pores, also called pseudocyphelae. This genus is the most productive of the Lobariaceae family in terms of the number of secondary metabolites. At the same time, it is more complex due to its chemical diversity [25], which makes it an interesting source for the study of bioactive compounds. *Pseudocyphellaria faveolata* (Delise) Malme is distributed in Chile from Nahuelbuta National Park (IX region) to Magallanes (XII region) [26].

In this study, the antimicrobial and antibiofilm activity of the secondary metabolites of Pseudocyphellaria faveolata (Delise) Malme were evaluated by the broth microdilution method. The safety of the more promising metabolite was also assessed on human fibroblasts. This study aimed to determine the antibacterial activity of Physciosporin and methylvirensate and the safety of the first in order to provide data for their further effective application in pharmaceuticals. 

## 2. Results

### 2.1. Chemistry

**Figure 1:**
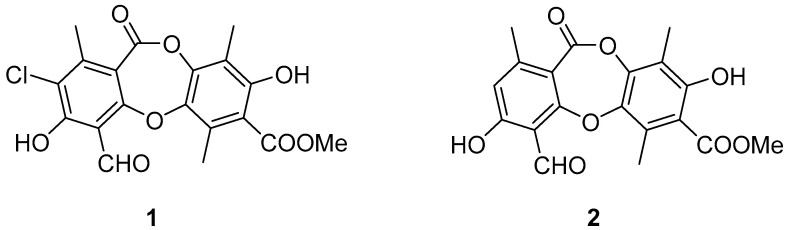
Metabolites of Pseudocyphellaria faveolata (Delise). Physciosporin (**1**); methyl virensate (**2**).

Physciosporin (**1**) and methyl virensate (**2**) were isolated from extract of Pseudocyphellaria faveolata (Delise) (Figure 1). These metabolites were purified by column chromatography and characterized by NMR spectroscopy and physical constants.

Physciosporin was obtained in yields of 6.14% as a pale yellow solid (mp 197.6–198.0 °C), and methyl virensate was obtained in yields of 0.75% as a white solid (mp 215.6–216.0 °C).

The spectroscopic assignment was carried out with experiments 1D (^1^HNMR and ^13^CNMR) and 2D (HMBC and HSQC). Molecule **1** in the ^1^HNMR exhibited signals for three acid protons (12.81, 11.41, and 10.16 ppm, respectively), one signal for the methyl ester (3.97 ppm), and three signals for methoxyl groups (2.60, 2.58 and 2.28 ppm, respectively); all signal were singlet. ^13^CNMR exhibited signals for three carbonyl groups (192.8, 171.3, and 162.7 ppm, respectively), twelve aromatic carbons (between 162.7 and 109.3 ppm), one methoxyl carbon (52.6 ppm), and three methoxyl carbons (19.8, 15.6, and 9.3 ppm).

Thanks to the correlation of the aldehyde proton signal (10.16 ppm) with the 161.3 and 160.0 ppm carbon signals and the correlation of the hydroxyl signal (12.81 ppm) with 162.3 ppm carbon signal, the 4-OH position was established. With correlation of the hydroxyl signal (11.41 ppm) with the 159.2 ppm carbon signal, the 2’-OH position was established. With these determinations, it was possible to establish the other carbons of the molecule through cross-peak *J*^3^ and *J*^2^. Only the methyls of positions 6 and 6’ could not be differentiated (interchangeable signals).

The spectroscopic data for molecule **2** are similar; however, an aromatic proton signal (6.69 ppm) was observed. This signal confirms the structure of methyl virensate. These data were compared and agreed with what was previously reported [14].

### 2.2. Antimicrobial Activity

**Figure 2:**
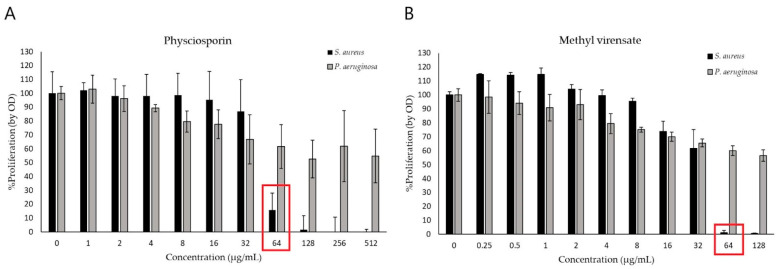
Effect of *Pseudocyphellarina faveolata* metabolites on bacterial proliferation. *Pseudomonas aeruginosa* (grey bars) and *Staphylococcus aureus* (black bars) were incubated for 24 h with (**A**) physciosporin and (**B**) methyl virensate. %Bacterial proliferation was determined by optical density compared to the untreated control (mean ± S.D.). The MIC of the studied compound on *S. aureus* is shown in red.

The antimicrobial activities of physciosporin (from 1 to 512 µg/mL) and methyl virensate (from 0.25 to 128 µg/mL) were studied in terms of their inhibitory, bactericidal, and antibiofilm activity on *Pseudomonas aeruginosa* and *Staphylococcus aureus* cultures. Figure 2 shows that both compounds had inhibitory effects on *S. aureus* proliferation, with an inhibitory activity starting from 64 µg/mL. None of the compounds significantly affected *P. aeruginosa* proliferation, showing a mild inhibition of about 40% at the highest concentrations tested.

**Figure 3:**
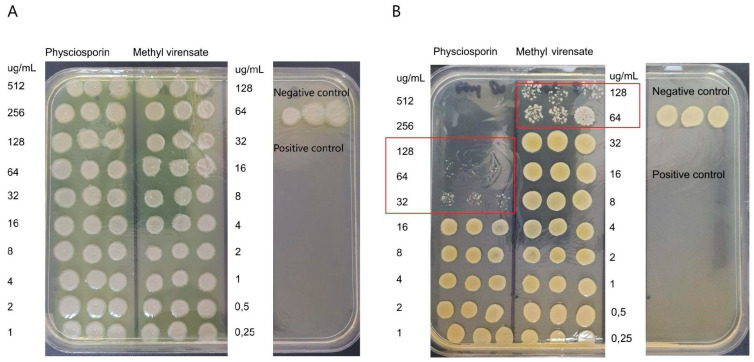
Effect of *Pseudocyphellarina faveolata* metabolites on bacterial viability. *Pseudomonas aeruginosa* (**A**) and *Staphylococcus aureus* (**B**) viability after treatment with physciosporin and methyl virensate. Red frames in the *S. aureus* plate highlight the concentration range where bacterial colonies are visibly reduced (MIC) or undetected (MBC). Positive controls are tobramycin sulfate (**A**) and chloramphenicol (**B**).

The effect of the compounds on viability was confirmed by plating the bacterial suspensions exposed to the treatments. Figure 3A shows a visible reduction in the number of *S. aureus* colonies at concentrations equal to or above 32 µg/mL of physciosporin (MIC). No viable colonies are observed at 128 µg/mL for physciosporin, indicating its MBC. On the other hand, methyl virensate showed a MIC of 64 µg/mL without an MBC identified at the tested concentrations. None of the compounds reduced *P. aeruginosa* viability (Figure 3B).

**Figure 4:**
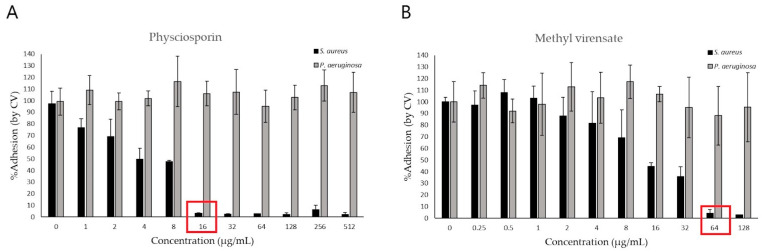
Effect of *Pseudocyphellarina faveolata* metabolites on bacterial adhesion. *Pseudomonas aeruginosa* (grey bars) and *Staphylococcus aureus* (black bars) were incubated for 24 h with (**A**) physciosporin and (**B**) methyl virensate. %Bacterial adhesion was determined by crystal violet (CV) staining compared to the untreated control (mean ± S.D.). Red squares show the concentration at which *S. aureus* biofilm formation was inhibited.

The antibiofilm activity of physciosporin and methyl virensate was studied by crystal violet staining to determine the remaining bacterial biomass adhered to the wells of the plates. Figure 4 shows these results as a percentage of adhesion compared to the untreated control. We can observe that both compounds can prevent *S. aureus* adhesion, with physciosporin completely inhibiting the adhesion at 16 µg/mL (Figure 4A) and methyl virensate at 64 µg/mL (Figure 4B). On the other hand, none of the compounds reduced *P. aeruginosa* adhesion at the studied concentration range.

**Figure 5:**
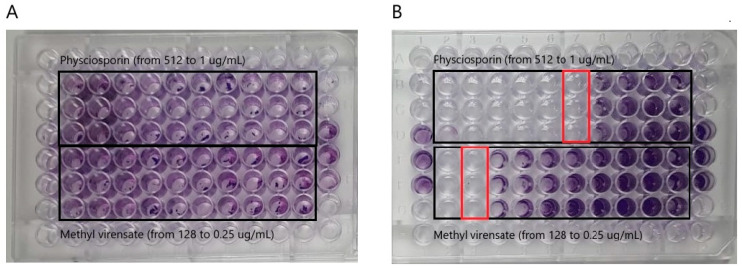
Effect of *Pseudocyphellarina faveolata* metabolites on bacterial adhesion. *Pseudomonas aeruginosa* (**A**) and *Staphylococcus aureus* (**B**) adhesion after treatment with physciosporin (from 1 to 512 µg/mL) and methyl virensate (from 0.25 to 128 µg/mL). Red frames in the *S. aureus* plate highlight the lowest concentration at which bacterial adhesion is visibly reduced or undetected, as measured by crystal violet staining.

Figure 5 shows pictures of representative plates on which crystal violet staining can visualize the adhered biomass remaining after the treatment. In the *S. aureus* plate (Figure 5B), a region with clear wells can be observed at the highest tested concentrations (i.e., no adhered bacteria are visible). In contrast, in the *P. aeruginosa* plate, all the wells seemed similarly stained without regions of clear wells.

### 2.3. Cell Viability

**Figure 6:**
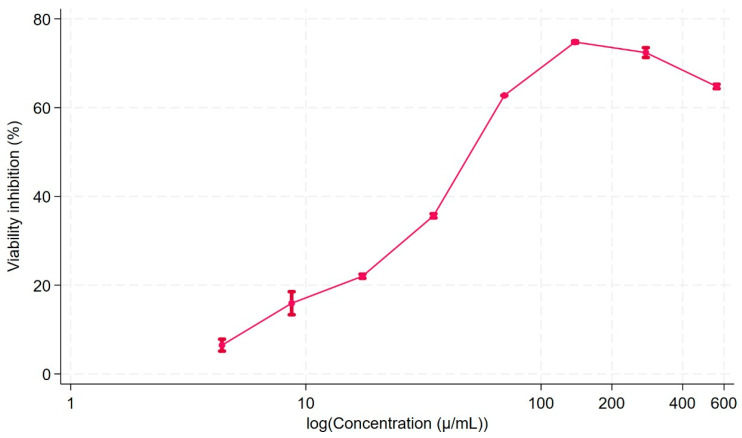
Viability inhibition of human normal fibroblasts by physciosporin. The assay was performed by seeding 5000 fibroblasts/well, and after 24 h, they were exposed to dilutions of physciosporin (558 to 4.4 µg/mL) for 48 h. Then, the cell culture media was replaced by resazurin 4 mg/L containing cell culture media for four hours and the fluorescence was read. The percentage of inhibition was calculated by comparison with DMSO (vehicle) exposed cells. The error bars correspond to the standard error.

Due to the promising results obtained with physciosporin, its cytotoxicity in normal human fibroblasts was determined by a metabolic viability assay based on the reduction of resazurin to a fluorescent compound when cells are viable [27]. In Figure 6, the viability inhibition curve is presented, showing the cytotoxicity exhibited by physciosporin. However, at 16 µg/mL a concentration that inhibits bacterial adhesion, there was about 20% viability inhibition, giving this molecule an interesting potential as anti-biofilm active principle that would not damage healthy animal bystander cells. The biofilm control is relevant in several biomedical applications, such as wound healing, dental caries, and catheter sanitization.

## 3. Discussion

From *Pseudocyphellaria faveolata* (Delise) Malme, physciosporin and methyl virensate were isolated and characterized by NMR spectroscopy. Compounds were obtained in yields of 6.14 and 0.75%, respectively. The percentage of yield for physciosporin is remarkable and allows us to project it for further research.

Since our research aims to find candidate molecules with potential for translation into in vivo studies, we focused on investigating isolated metabolites. While whole-natural extracts are often used in traditional medicine with promising results, their lack of standardization poses significant challenges for clinical translation and commercialization. Focusing on isolated compounds allows for precise characterization and facilitates further development towards therapeutic applications. Additionally, the use of whole natural extracts for product development is often constrained by the availability of sufficient biomass, which is lichen material. Lichens grow slowly and are usually found in limited quantities, making large-scale harvesting unsustainable and impractical for commercial purposes. By focusing on isolated compounds, we not only mitigate the dependency on extensive biomass collection but also gain the ability to explore potential structural modifications or synthetic routes. This approach allows us to optimize the bioactive properties of the compounds and enhance their scalability, stability, and suitability for therapeutic development.

### Antimicrobial Activity of Physciosporin and Methyl Virensate

Both metabolites completely inhibited *S. aureus* proliferation (Figure 2), while in contrast, the effect on *P. aeruginosa* proliferation was modest (about 50% inhibition at the highest tested concentrations). Physciosporin also had bactericidal and antibiofilm activities against *S. aureus* (Figure 3 and Figure 5).

Inhibition of bacterial adhesion is a relevant activity as it can prevent the formation of highly resistant microbial aggregates known as biofilms, with a lower potential for developing resistance than conventional bactericidal or bacteriostatic antibiotics [5]. Biofilm formation begins from the first adhesion of colonizing bacteria that proliferate in a sessile state and produce a polymeric extracellular substance that protects the community from environmental factors [28]. These biofilm infections are developed in patients with underlying chronic diseases that compromise their immune response. Some examples are biofilms found in chronic wounds of diabetic patients, lung infections in cystic fibrosis patients, or catheter-associated urinary tract infections [4]. *S. aureus* is a relevant human pathogen and one of the most prevalent microorganisms in biofilm infections related to medical devices. These infections include valvular prosthesis endocarditis, infections associated with total joint arthroplasty, and intravascular catheter-related infections [29]. Therefore, novel compounds are urgently needed to treat these infections and prevent their establishment. Among the studied compounds, physciosporin represents a promising antimicrobial candidate demonstrating the potential to inhibit adhesion and, consequently, prevent the formation of biofilms below its MIC and MBC. Further studies involving in vivo models of biofilm infections, particularly those associated with medical devices, are needed to validate its efficacy.

The literature shows few reports on methyl virensate, with nothing evaluating its biological activities. On the other hand, some reports on the activities of physciosporin can be found. These reports from the same research group described the antiproliferative activity of physciosporin isolated from *Pseudocyphellaria granulata* or *coriacea* against lung [30], colon [31], and breast [32] cancer cells. Physciosporin decreased viability, induced apoptosis, and inhibited migration and invasion through novel mechanisms, including a reduction in the expression of N-cadherin. This antitumoral activity was also observed in vivo without toxicity. Though the antimicrobial properties of lichen extracts and compounds have been described [33], to our knowledge, this is the first report on the antimicrobial properties of physciosporin and methyl virensate.

These results are interesting and allow us to propose the study of new chlorinated depsidones such as norpannarin and vicanicin which have not yet been studied as antimicrobials. On the other hand, the yield of physciosporin allows it to be a suitable scaffold for subsequent structural modifications that improve its activity by modifying its physicochemical and pharmacokinetic properties.

Most studies investigating the antibacterial activity of secondary metabolites do not evaluate their cytotoxicity on normal human animal cells. Here, we performed such a study using commercial normal fibroblasts, the most abundant cells in the human body, conforming the connective tissue, that would be exposed to the molecule if an in vivo administration occurred. We showed cytotoxicity on fibroblasts, consistent with the effects reported on cancer cells [30,31,32]. Interestingly, at the concentration that showed anti-biofilm activity, there was an acceptable inhibition of the normal cell viability. We are here presenting a comprehensive work in the search for new antibiotics, evaluating the antimicrobial activity while considering the cytotoxicity assessment of the eukaryotic cells. In other words, an antibiotic candidate should be toxic to bacteria but not to the hosts’ cells.

## 4. Materials and Methods

### 4.1. Lichen Material

Thalli of *Pseudocyphellaria faveolata* (Delise) Malme were collected randomly in Conguillio (IX Region), Chile, in January 2020. Representative specimens were deposited in the Lichen Herbarium of the Escuela de Química y Farmacia, Universidad de Valparaiso. The taxonomic determination was carried out following dichotomous keys and comparing herbarium samples. A stereoscopic magnifying glass (ZEISS Stemi 305, Oberkochen, Germany) was used to observe morphological features.

### 4.2. Spectroscopic Analysis

^1^HNMR and ^13^CNMR spectra were recorded in CDCl_3_ and are referenced to the residual peaks of CHCl_3_ at δ = 7.26 ppm and δ = 77.0 ppm for ^1^H and ^13^C, respectively, on an Avance 400 digital NMR spectrometer (Bruke, Rheinstetten, Germany) operating at 400.1 MHz for ^1^H and 100.6 MHz for ^13^C.

### 4.3. Isolation of Compounds

Physciosporin (**1**) and methyl virensate (**2**) were isolated from the extract of *Pseudocyphellaria faveolata* (Delise) Malme. The lichen material was extracted in solvents of increasing polarity (hexane, ethyl acetate, and acetone). The extracts were concentrated under reduced pressure. The extracts were compared by TLC and the same two products were observed in all of them. The extract was separated by column chromatography, and two pure products were obtained; this compound was characterized by melting point (Stuart SMP30) and identified by spectroscopic data (^1^HNMR, ^13^CNMR, HMBC, HSQC).

Physciosporin (C_19_H_15_ClO_8_): pale yellow solid (mp 197.6–198.0 °C).

^1^H-RMN (CDCl_3_, 400 MHz) δ: 12.81 (1H, s, OH); 11.41 (1H, s, OH); 10.16 (1H, s, CHO); 3.97 (3H, s, COOMe); 2.60 (3H, s, Me); 2.58 (3H, s, Me); 2.28 (3H, s, Me).

^13^C-RMN (CDCl_3_, 100 MHz) δ: 192.8 (C8-CHO); 171.3 (C7’); 162.7 (C7); 161.3 (C4); 161.0 (C2); 159.3 (C2’); 150.6 (C1); 146.8 (C4’); 142.4 (C5’); 129.2 (C6’); 121.3 (C5); 117.2 (C3’); 114.3 (C6); 110.8 (C3); 109.3 (C1’); 52.6 (COOMe); 19.8 (Me)*; 15.6 (Me)*; 9.3 (C3’-Me). * interchangeable signals.

Methyl virensate (C_19_H_16_O_8_): white solid (mp 215.6–216.0 °C)

^1^H-RMN (CDCl_3_, 400 MHz) δ: 12.18 (1H, s, OH); 11.44 (1H, s, OH); 10.70 (1H, s, CHO); 6.69 (1H, s, H5); 3.96 (3H, s, COOCH_3_); 2.57 (3H, s, Me); 2.52 (3H, s, Me); 2.26 (3H, s, Me).

^13^C-RMN (CDCl_3_, 100 MHz) δ: 194.0 (C8-CHO); 171.4 (C7’); 165.6 (C7); 165.0 (C4); 161.2 (C2); 159.2 (C2’); 154.0 (C1); 146.9 (C4’); 142.4 (C5’); 129.2 (C6’); 117.6 (C5); 117.1 (C3’); 112.7 (C6); 110.8 (C3); 109.3 (C1’); 52.6 (COOMe); 22.3 (Me)*; 14,1 (Me)*; 9.3 (C3’-Me). * interchangeable signals.

### 4.4. Bacterial Culture

*Pseudomonas aeruginosa* (ATCC 27853) and *Staphylococcus aureus* (ATCC 29213) were obtained from Microbiologics Inc., St. Cloud, MN, United States) and maintained in frozen stocks in 50% *v*/*v* glycerol in culture media. *P. aeruginosa* was cultured in LB agar or broth, and *S. aureus* in tryptic soy agar (TSA) or broth (TSB). Agar plates were inoculated with bacteria from the frozen stocks and incubated overnight at 37 °C in a static incubator (Heratherm IGS60, Thermo Fisher Scientific, Columbus, OH, USA). Then, about five isolated colonies of *P. aeruginosa* and *S. aureus* were used to inoculate centrifuge tubes with broth, and these tubes were incubated at 37 °C and 180 rpm in an orbital incubator (MaxQ4000, Thermo Fisher Scientific, Columbus, OH, USA). After overnight culture, bacterial suspensions were centrifuged, rinsed with PBS, and adjusted with broth to 3 × 10^7^ CFU/mL by spectrophotometry (at 600 nm, Synergy H1M, BioTek Instruments, Santa Clara, CA, USA).

### 4.5. Determination of Minimum Inhibitory (MIC) and Bactericidal Concentration (MBC)

The antimicrobial properties of the metabolites were tested using the broth microdilution method [34]. Briefly, metabolite stock solutions of physciosporin (18.6 mg/mL) and methyl virensate (5.2 mg/mL) were prepared in 100% *v*/*v* dimethyl sulfoxide (DMSO). Then, these stock solutions were diluted 16 times in culture broth (LB for *P. aeruginosa* and TSB for *S. aureus*) to achieve a DMSO concentration equal or below 6%. These solutions were serially two-fold diluted to obtain 9 different concentrations with final volumes of 100 µL within flat bottom tissue culture-treated 96-well plates. Culture media was used as a negative control, and chloramphenicol (16 µg/mL) and tobramycin sulfate (1 µg/mL) were used as a positive control for *S. aureus* and *P. aeruginosa*, respectively. DMSO was also tested at the same final concentrations. The adjusted bacterial suspensions (described above) were diluted 100x, combined at equal volumes with the treatments, and incubated at 37 °C and 180 rpm for 24 h before recording the absorbance at 625 nm as a measurement of bacterial proliferation. Sterile treatment absorbance blanks were also included. Each concentration was tested in triplicate in each independent experiment (n = 2 or 3).

For MIC and MBC determination, the treated bacterial suspensions from the broth microdilution were seeded in agar plates using the drop plate method [35]. Drops of 10 µL of each treatment concentration were seeded in 100 mm squared agar plates and incubated at 37 °C overnight. The lowest concentration at which the number of colonies was visibly reduced was the MIC, and the lowest concentration at which no viable colonies were observed was the MBC.

### 4.6. Determination of Antibiofilm Activity

Crystal violet staining was used to determine the effect of the metabolites on *P. aeruginosa* and *S. aureus* adhesion and biofilm formation, as described by [36]. Briefly, the wells of the plates from the broth microdilution were rinsed with distilled water to remove free-floating bacteria and air-dried over. The wells were incubated for 30 min at room temperature with 200 μL of methanol to dehydrate and fix the microbial growth adhered to the well surface. Then, methanol was discarded, and the plate was allowed to air-dry overnight before adding 200 μL of 0.1% *w*/*v* crystal violet. After 15 min at room temperature, the wells were rinsed with distilled water and air-dried. Finally, the retained crystal violet dye was dissolved with 30% *v*/*v* acetic acid, and the absorbance was recorded at 570 nm.

### 4.7. Cell Viability Assay

Commercial human normal fibroblasts were acquired from Gibco (HDFn, C0045C, Thermo Fischer Scientific, Grand Island, NY, USA). The viability of the cells exposed to physciosporin was measured using the resazurin assay [27]. This method is based on the ability of viable cells to reduce this molecule to form a fluorescent product with an excitation wavelength at 544 nm and an emission at 590 nm. Viability was calculated from the ratio of the difference of relative fluorescence units (RFU) for cells grown in vehicle control and the cells exposed to the treatment versus the fluorescence of the cells grown in vehicle control media, expressed as a percentage. Therefore, to achieve our objective of evaluating the cytotoxicity of physciosporin, 5000 fibroblasts were seeded on 96-wells plate, after cell-adhesion, the cell cultures were exposed to dilutions of physciosporin in a range of 4.4 to 558.0 µg/mL or vehicle control (DMSO dilutions). After 48 h, the cell culture was removed and resazurin 4 mg/L in cell culture media was added for four h, and the fluorescent was read in a Varioscan Thermofischer Scientific plate reader (Brisbane, Australia). Each condition was tested in triplicate and three independent experiments were performed.

## 5. Conclusions

This is the first report on the study of the antimicrobial activity of physciosporin and methyl virensate. Both metabolites completely inhibited *S. aureus* proliferation. Physciosporin also had bactericidal (32 µg/mL, MIC) and antibiofilm activities against *S. aureus.* Physciosporin showed promising activity in preventing the formation of *S. aureus* biofilms, which are responsible for chronic infections; these activities occurred at a concentration with low cytotoxicity on normal human cells. These findings provide a foundation for exploring the antimicrobial potential of other lichenic depsidones or for investigating physciosporin as a basic structure to develop new compounds with antimicrobial potential and limited toxicity on animal cells.

## Data Availability

The original contributions presented in this study are included in the article/supplementary material. Further inquiries can be directed to the corresponding authors.

## Supplementary Materials

The following supporting information can be downloaded at: https://www.mdpi.com/article/10.3390/molecules30061368/s1, NMR spectroscopy. Figure S1: The ^1^H NMR spectrum of compound **2**, Figure S2: The ^13^C NMR spectrum of compound **2**, Figure S3: The Dept 135 NMR spectrum of compound **2**, Figure S4: The HMBC NMR spectrum of compound **2**, Figure S5: The HSQC NMR spectrum of compound **2**, Figure S6: The ^1^H NMR spectrum of compound **1**, Figure S7: The ^13^C NMR spectrum of compound **1**, Figure S8: The Dept 135 NMR spectrum of compound **1**, Figure S9: The Dept 135 HMBC NMR spectrum of compound **1**.
