# Supplementary material for: Isolation, Chemical Characterization, and Antimicrobial Activity of Secondary Metabolites from Pseudocyphellaria faveolata
Source: Molecules. 2025 Mar 18;30(6):1368. doi: 10.3390/molecules30061368 (PMC11944333; doi:10.3390/molecules30061368)
Supplement: Supplementary file 1 [file molecules-30-01368-s001.zip › molecules-3379933-supplementary.pdf]

Isolation, Chemical Characterization, and Antimicrobial Activity of Secondary Metabolites from *Pseudocyphellaria faveolata*

Cecilia Rubio [1,2], Javiera Ramírez [1], Caroline Weinstein-Oppenheimer [3,4], Tania F. Bahamondez-Canas [4,5] and Natalia Quiñones [1,4,*]

[1] Herbario de Líquenes, Escuela de Química y Farmacia, Facultad de Farmacia, Universidad de Valparaíso, Valparaíso 2340000, Chile; cecilia.rubio-@uv.cl (C.R.); javiera.rp6@gmail.com (J.R.)
[2] Magíster en Gestión Farmacéutica y Farmacia Asistencial, Escuela de Química y Farmacia, Facultad de Farmacia, Universidad de Valparaíso, Valparaíso 2340000, Chile
[3] Laboratorio de Innovación Terapéutica y Diagnóstico Molecular, Escuela de Química y Farmacia, Facultad de Farmacia, Universidad de Valparaíso, Valparaíso 2340000, Chile; caroline.weinstein@uv.cl
[4] Centro de Investigación, Desarrollo e Innovación de Productos Bioactivos (CInBIO), Universidad de Valparaíso, Valparaíso 2340000, Chile; tania.bahamondez@uv.cl
[5] Laboratorio de Farmacotecnia Antimicrobiana (LADEFAM), Escuela de Química y Farmacia, Facultad de Farmacia, Universidad de Valparaíso, Valparaíso 2340000, Chile
* Correspondence: natalia.quinones@uv.cl

Supporting Information

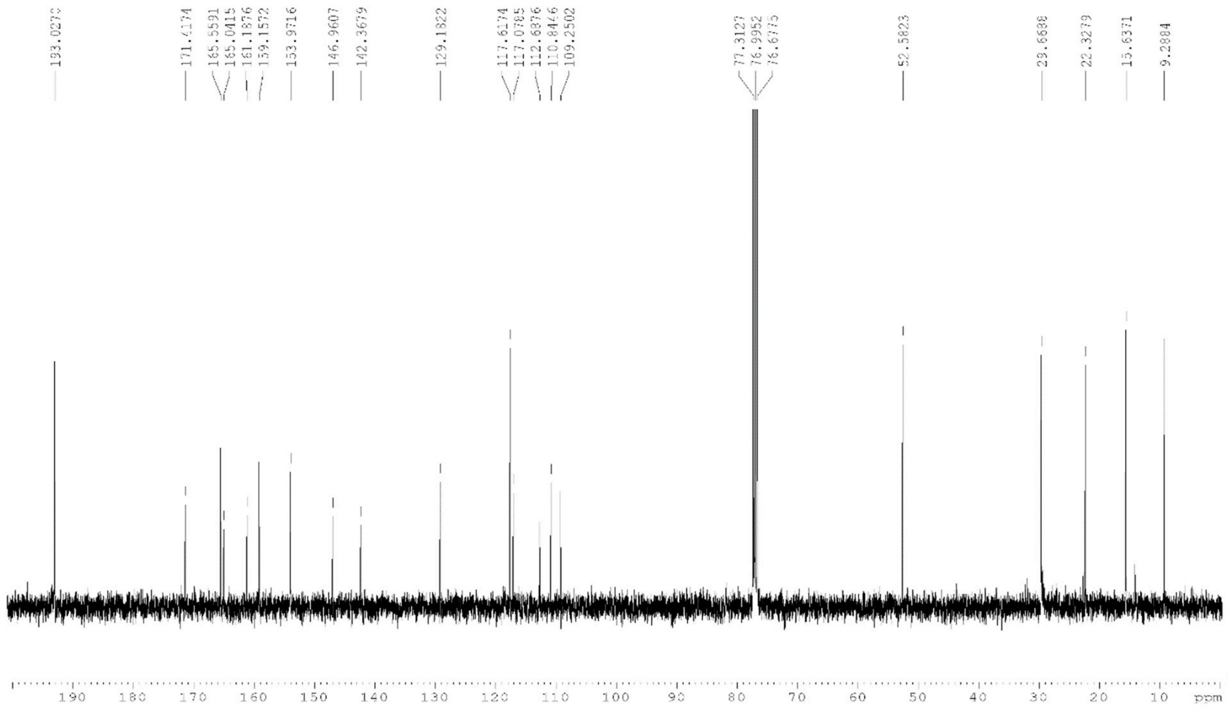

Figure S1: The ^{1}H NMR spectrum of compound **2.**

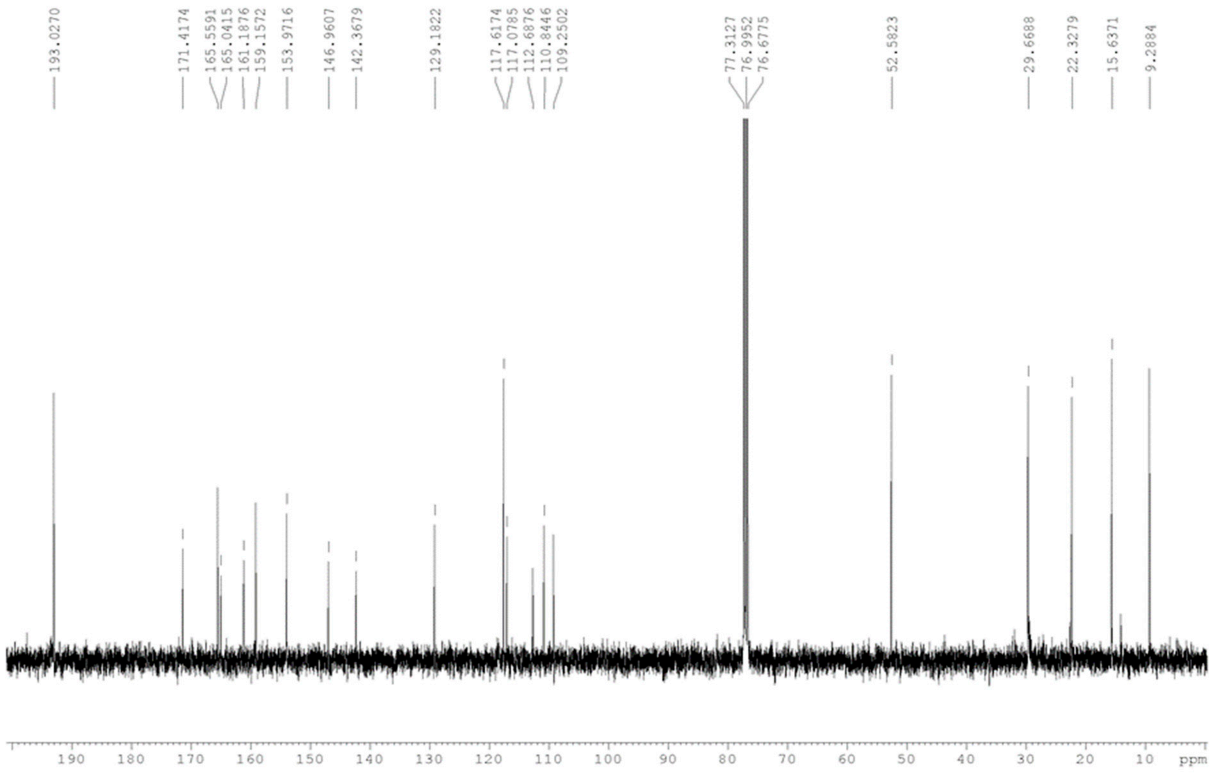

Figure S2: The ^{13}C NMR spectrum of compound **2.**

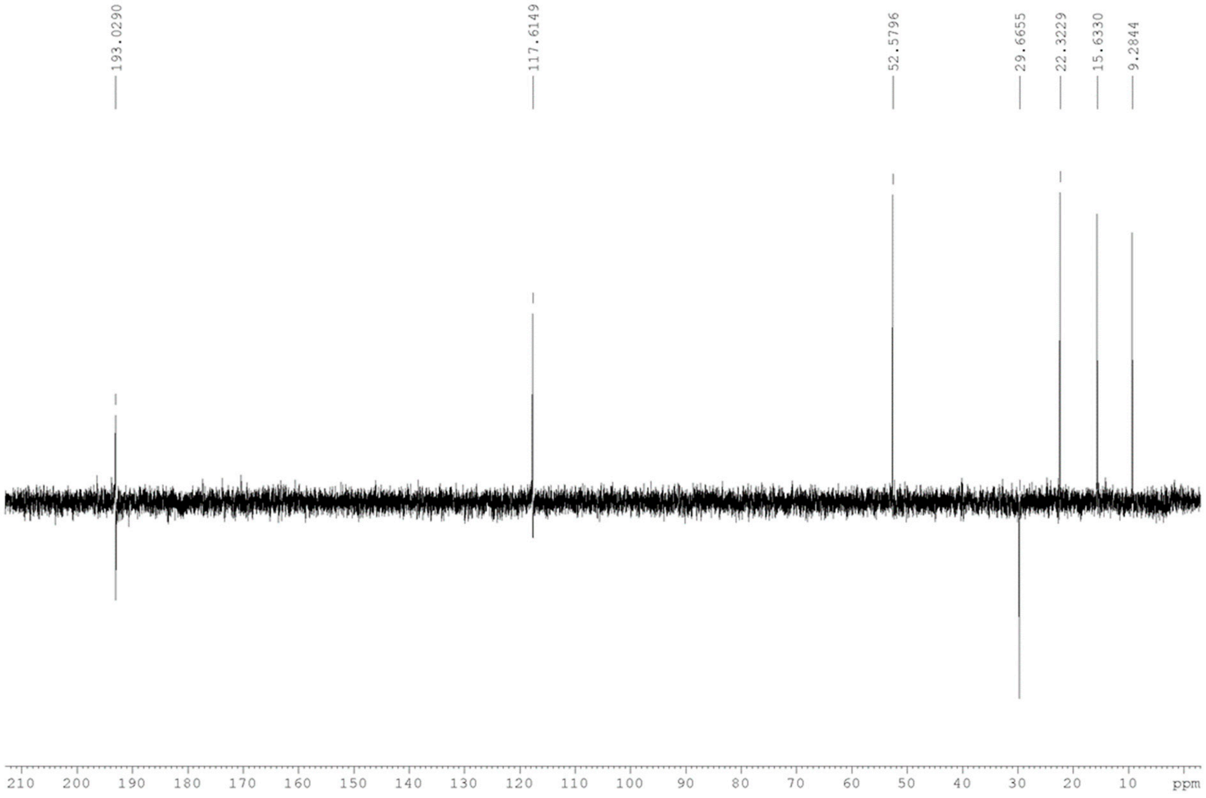

Figure S3: The Dept 135 NMR spectrum of compound **2.**

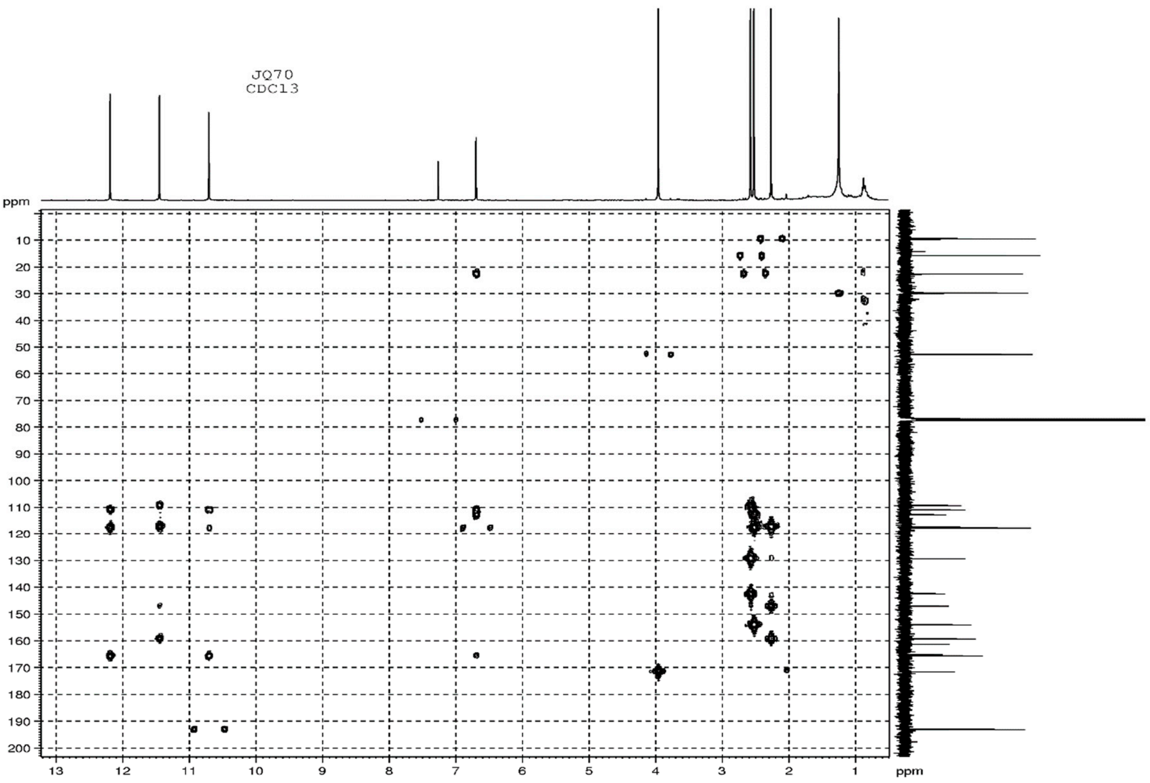

Figure S4: The HMBC NMR spectrum of compound **2**.

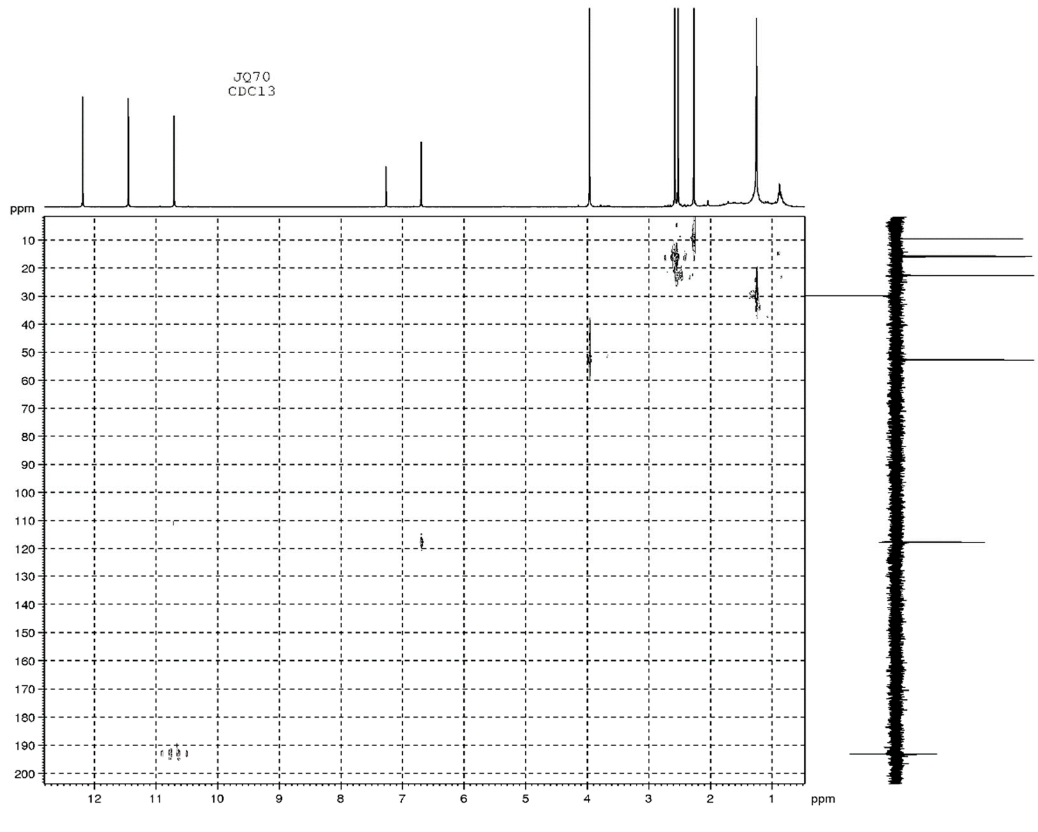

Figure S5: The HSQC NMR spectrum of compound **2.**

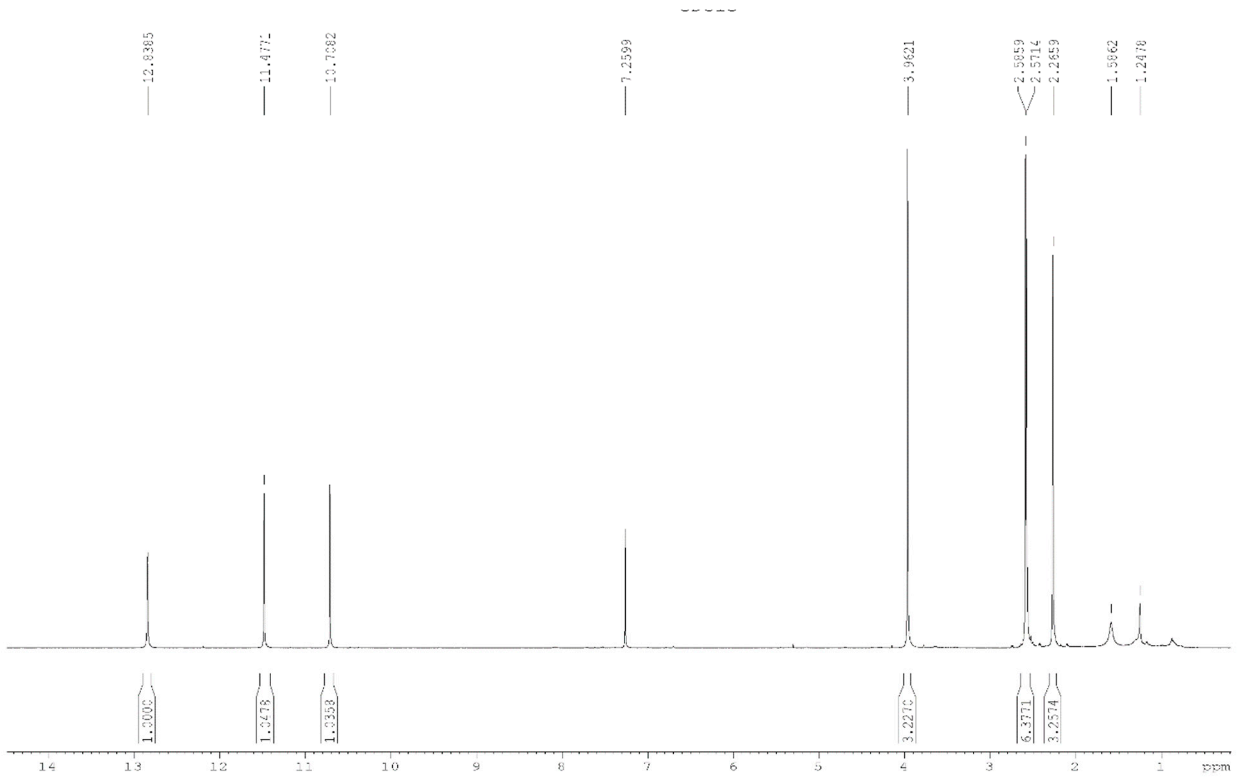

Figure S6: The ^{1}H NMR spectrum of compound **1.**

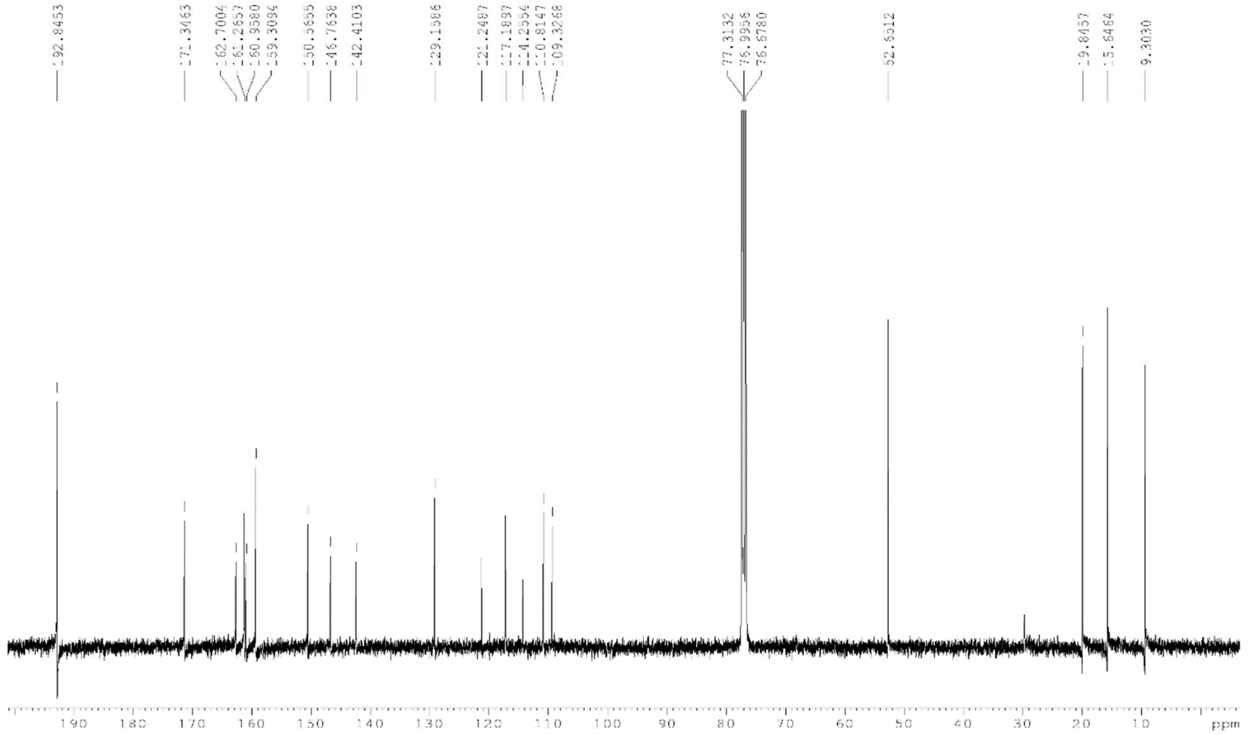

Figure S7: The ^{13}C NMR spectrum of compound **1**.

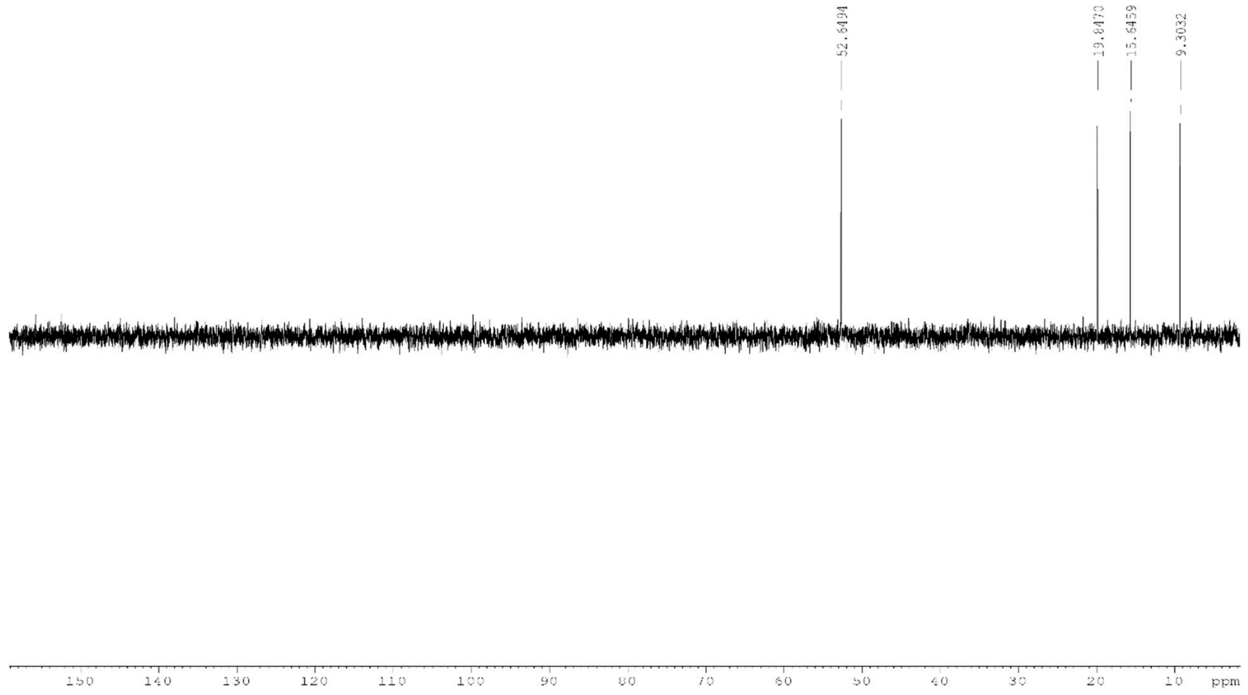

Figure S8: The Dept 135 NMR spectrum of compound **1.**

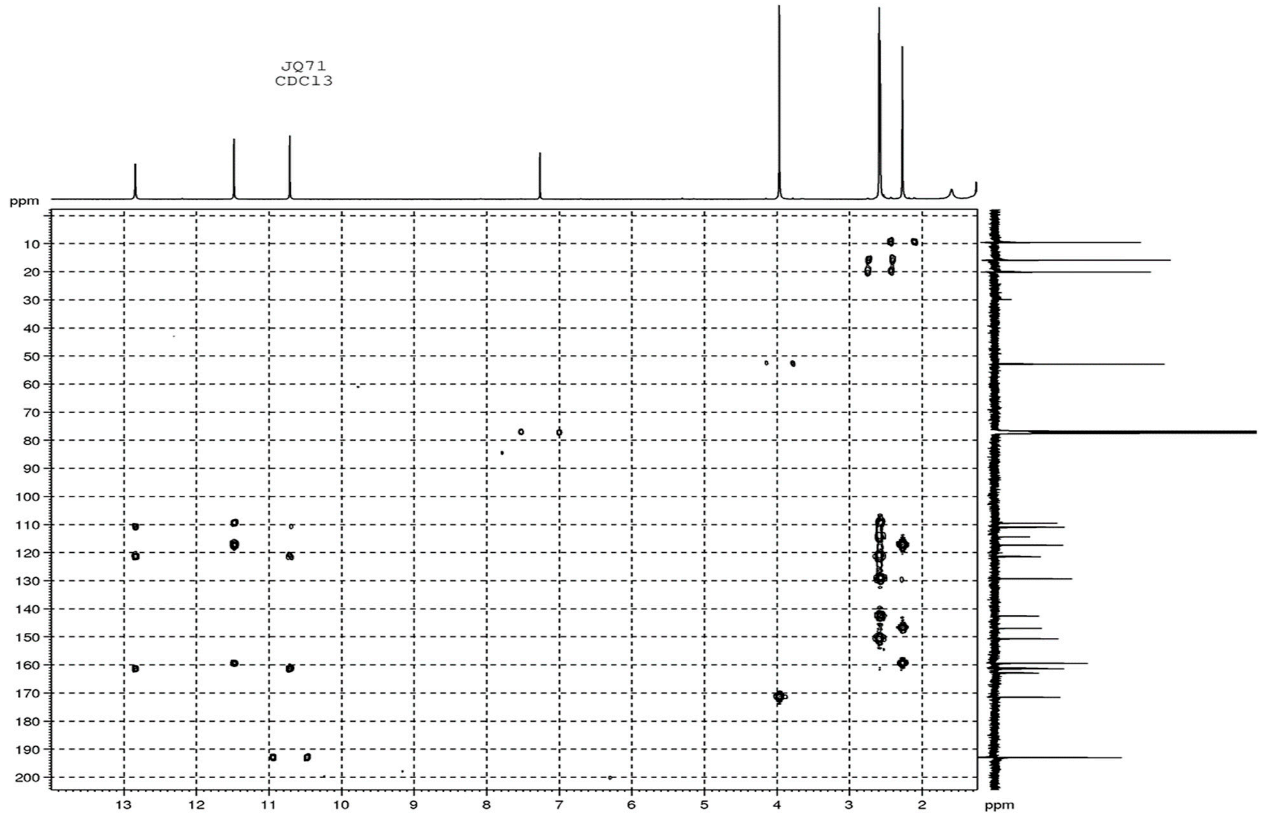

Figure S9: The Dept 135 HMBC NMR spectrum of compound **1.**
